# RAF Kinase Activity Regulates Neuroepithelial Cell Proliferation and Neuronal Progenitor Cell Differentiation during Early Inner Ear Development

**DOI:** 10.1371/journal.pone.0014435

**Published:** 2010-12-28

**Authors:** Marta Magariños, María R. Aburto, Hortensia Sánchez-Calderón, Carmen Muñoz-Agudo, Ulf R. Rapp, Isabel Varela-Nieto

**Affiliations:** 1 Instituto de Investigaciones Biomédicas “Alberto Sols”, CSIC-UAM, Madrid, Spain; 2 Departamento de Biología, Universidad Autónoma de Madrid, Madrid, Spain; 3 Unit 761, Centro de Investigación Biomédica en Red de Enfermedades Raras (CIBERER), Instituto de Salud Carlos III, Madrid, Spain; 4 Department of Molecular Biology, Max-Planck-Institute of Biochemistry, Munich, Germany; Universidade Federal do Rio de Janeiro, Brazil

## Abstract

**Background:**

Early inner ear development requires the strict regulation of cell proliferation, survival, migration and differentiation, coordinated by the concerted action of extrinsic and intrinsic factors. Deregulation of these processes is associated with embryonic malformations and deafness. We have shown that insulin-like growth factor I (IGF-I) plays a key role in embryonic and postnatal otic development by triggering the activation of intracellular lipid and protein kinases. RAF kinases are serine/threonine kinases that regulate the highly conserved RAS-RAF-MEK-ERK signaling cascade involved in transducing the signals from extracellular growth factors to the nucleus. However, the regulation of RAF kinase activity by growth factors during development is complex and still not fully understood.

**Methodology/Principal Findings:**

By using a combination of qRT-PCR, Western blotting, immunohistochemistry and in situ hybridization, we show that C-RAF and B-RAF are expressed during the early development of the chicken inner ear in specific spatiotemporal patterns. Moreover, later in development B-RAF expression is associated to hair cells in the sensory patches. Experiments in ex vivo cultures of otic vesicle explants demonstrate that the influence of IGF-I on proliferation but not survival depends on RAF kinase activating the MEK-ERK phosphorylation cascade. With the specific RAF inhibitor Sorafenib, we show that blocking RAF activity in organotypic cultures increases apoptosis and diminishes the rate of cell proliferation in the otic epithelia, as well as severely impairing neurogenesis of the acoustic-vestibular ganglion (AVG) and neuron maturation.

**Conclusions/Significance:**

We conclude that RAF kinase activity is essential to establish the balance between cell proliferation and death in neuroepithelial otic precursors, and for otic neuron differentiation and axonal growth at the AVG.

## Introduction

The vertebrate inner ear is responsible for the detection of sound and balance, and it contains two main functional parts, the auditory system dedicated to hearing and the vestibular system that controls balance. This complex sensory organ derives from an ectodermic region adjacent to the hindbrain, the otic placode. As development proceeds, the otic placode thickens, invaginates and forms the otic cup, which will then close to form an ectoderm-detached, pear-shaped structure: the otic vesicle or otocyst [1]. The otic vesicle is an autonomous structure that contains the genetic information required to generate most of the cell types and structures of the adult inner ear, including the neurons of the acoustic-vestibular ganglion (AVG) [2], [3]. The AVG contains the neural precursors of the auditory and vestibular ganglia, which form a single ganglion at this stage of development. The neurons involved are specified in the otic epithelium and these neuroblasts migrate from the neurogenic zone to a nearby area where, after an intense period of proliferation, they differentiate into post-mitotic neurons that extend their processes to the sensory epithelium in the brainstem nuclei through the VIIIth cranial nerve [1], [2], [4], [5].

Otocysts can be explanted from the embryo and their *ex vivo* development can be followed in a defined culture medium to study the molecular cues that instruct the cellular diversity found *in vivo* [4]. Through the combination of *in vivo* and organotypic culture studies, it has been shown that Wnt, fibroblast growth factors, neurotrophins and factors of the insulin family can reinitiate cell proliferation of quiescent otic vesicles, to drive morphogenesis, determine cell fate specification, and promote migration or final differentiation [6]–[9].

Insulin-like growth factor I (IGF-I) has been shown to modulate otic development in evolutionary distant species [4] and indeed, IGF-I deficit is associated to profound sensorineural deafness and cochlear malformation in man and mice (MIM 147440) [10], [11]. IGF-I deficit in the mouse is associated with caspase-3-mediated apoptosis of immature cochlear neurons [12] and with altered signaling pathways, including poor activation of Akt and ERK1/2, and the up-regulation of p38 kinase pathways [13]. Cochlear ganglion neurons have many immature traits including the aberrant expression of the MEF2A, MEF2D, SIX 6 and MASH1 transcription factors [13]. In the chicken inner ear, IGF-I drives cellular programs that are important for specific events during otic development, including proliferation, survival, metabolism and differentiation [7]. Both IGF-I and its high affinity IGF1R receptor are expressed during inner ear development [6]. Moreover, endogenous otic IGF-I activity is essential for the survival and neurogenesis of otic precursors due to its activation of the PI3K/Akt kinase pathway [6], [14]. On the other hand, exogenous IGF-I mimics morphogenetic traits in vivo, promoting neurogenesis and axon sprouting, accelerating the rate of cell proliferation and improving cell survival by inhibiting apoptosis of both epithelial and neural progenitors [6]. IGF-I can activate the RAF-MEK-ERK cascade in the otic epithelium, and C-RAF is essential for otic vesicle proliferation and morphogenesis [15]. However, it is not still fully clear how the strict balance between signaling pathways is regulated by IGF-I during development.

RAF kinases are serine/threonine kinases whose activity is modulated by growth factors and that play a central role in normal and pathologic cellular processes, including development, cell regeneration, cell senescence and cancer [16]. The first RAF kinase identified was the oncogenic product of mouse sarcoma virus 3611 [17] and since, the mammalian RAF kinases have been shown to belong to a family that is formed by A-, B- and C-RAF. In invertebrates only a single RAF kinase exists whereas the two isoforms in birds are homologues of B- and C-RAF [18]. In mammals, A-RAF is the less abundant kinase and it is expressed in the urogenital and gastrointestinal systems. By contrast, B-RAF is more abundant and it is found in the nervous system and gonads, whereas C-RAF is ubiquitously expressed [19]. The study of knock-out mice lacking each of these kinases has revealed that they fulfill common and distinct functions [18], [20], as well as shedding light on how they are regulated, their distinct intracellular localization [21] and association with scaffold proteins [22]. Indeed, B-RAF knock-out mice have defects in neural cell lineages, including reduced cell proliferation in the neocortex, and impaired migration and dendrite formation associated with cortical neurons [20].

RAF kinases transmit growth factor signals from the receptor/RAS complex via the phosphorylation of MEK in the cytosol. This leads to the phosphorylation and activation of ERK that in turn, can phosphorylate cytoplasmic and nuclear transcription factors that regulate gene expression and cellular responses. Bcl-2 family members are targets of the RAF-MEK-ERK pathway [23], and therefore, RAF kinases are considered to be anti-apoptotic factors. The activation of the RAS-RAF-MEK-ERK cascade is essential for cellular proliferation during malignant transformation [16], which has led to the synthesis of bi-aryl urea Sorafenib that inhibits the catalytic activity of B-RAF and C-RAF and that also blocks proangiogenic-receptor-tyrosine kinases [24]. B-RAF is the most potent of the kinases that phosphorylates ERK [25] and it is strongly expressed in the nervous system [26], [27]. However, its expression during inner ear development and the participation of RAF kinases in AVG neurogenesis has not yet been explored.

Here we show that both B-RAF and C-RAF are present in the inner ear during its early development *in vivo*, and that B-RAF expression becomes restricted as development proceeds. IGF-I can activate the RAF-MEK-ERK cascade in explanted otic vesicles and by blocking RAF kinases with Sorafenib, we show that RAF activity is essential for cell proliferation. By contrast, survival may be recovered by IGF-I induction of the PI3K/Akt kinase pathway. Finally, our data show that the RAF-MEK-ERK cascade is an important mediator of otic neuronal survival, migration and the outgrowth of neuronal processes.

## Materials and Methods

### Ethics Statement

All animals were handled in strict accordance with good animal practice as defined in the European Council Directive (86/609/EEC), and all animal work was approved by the Ethics Committee of the UAM and the Bioethics Committee of the *Consejo Superior de Investigaciones Cientificas*.

### Chicken embryos

Chicken embryos were obtained from fertilized eggs from a local farm (Granja Santa Isabel, Cordoba, Spain) and they were incubated in a humidified atmosphere at 37.8°C. Embryos were staged as HH18, HH20, HH22, HH24, HH27 and HH34 according to Hamburger and Hamilton's criteria [28].

### Isolation, organotypic culture and treatment of otic vesicles and AVG

Embryos at stage HH18 (65 h of incubation) were obtained and the otic vesicles were dissected from the surrounding mesenchymal tissue with sharpened tungsten needles, they were transferred into four-well culture-plates (Nunc, Roskilde, Denmark) and then incubated at 37°C in a water-saturated atmosphere containing 5% CO_2_, as described previously [7]. The standard culture medium consisted of M199 medium with Earle's salts (Sigma-Aldrich, Saint Louis, MO) supplemented with 2 mM glutamine (Gibco, Paisley, UK) and antibiotics [50 IU/ml penicillin (Ern, Barcelona, Spain) and 50 µg/ml streptomycin (CEPA, Madrid, Spain)]. AVG were obtained from stage HH19^+^ chicken embryos dissected out aseptically and plated onto glass coverslips that had been previously coated with poly-D-lysine and fibronectin [5]. The AVG was cultured in 0.25 ml F12/Dulbecco's modified Eagle medium (Gibco) containing 100 µg/ml transferrin, 16 µg/ml putrescine, 6ng/ml progesterone, 5.2 ng/ml sodium selenite (all from Sigma), and antibiotics as above.

Explanted otic vesicles were treated with IGF-I (10 nM, Recombinant IGF-I Roche Molecular Biochemicals, Basel, Switzerland), various concentrations of Sorabenib (BAY 43-9006 1, 5 and 10 µM; Bayer HealthCare Pharmaceuticals, West Haven, CT, USA), the MEK inhibitor U0126 (50 µM; Promega, Madison, WI), the C-RAF inhibitor GW5074 (1 µM; Sigma-Aldrich, Saint Louis, MO) the PI3K inhibitor LY294002 (25 µM; Cell Signaling Boston, MA) or a pan-caspase inhibitor Boc-D-FMK (100 µM; Calbiochem La Jolla, CA) for the times indicated in the text. The solvent used (DMSO) had no detectable effect on cultured otic vesicles when used at a final concentration of 0.01% for LY294002, Boc-D-FMK and Sorafenib cultures, and 0.2% for U0126 cultures. Otic vesicles cultured in medium without additives were used as controls (0S). For immunostaining and TUNEL labeling otic vesicles were fixed for 2 h in 4% (w/v) paraformaldehyde (Merck, Darmstadt, Germany) at 4°C. When indicated, the otic vesicle and AVG areas were measured using Image Analysis Software (Olympus, Tokyo, Japan). At least five explants per condition were assayed from 2–6 independent experiments and the statistical significance was estimated using the Student's t-test.

### Quantitative RT-PCR

Inner ears from chicken embryos were pooled to obtain RNA at different stages: HH18 (n = 40), HH22 (n = 25), HH24 (n = 20) and HH27 (n = 10). Three independent RNA pools from each stage were isolated with Trizol (Invitrogen) following the manufacturer's instructions, and the integrity and concentration of the RNA was assessed with an Agilent Bioanalyzer 2100 (Agilent Technologies). From this RNA, cDNA was generated by reverse transcription (High Capacity cDNA Reverse Transcription Kit: Applied Biosystems). Real-Time PCR of each pool was performed in triplicate using specific oligonucletides from “Quantitec Primer Assays” for chicken *B-Raf* and *C-Raf* (Gg_BRAF_1_SG (QT01141413), Gg_RAF1_1_SG (QT00599123); Geneglobe, Qiagen) and using SYBR Green as the detection system. PCR was performed on an Applied Biosystems 7900HT Real-Time PCR System using eukaryotic 18S rRNA as the endogenous housekeeping gene (Hs99999901_s1, TaqMan, Applied Biosystems). The estimated gene expression was calculated as 2^−ΔΔCt^ and statistical significance was estimated using the Student's t-test.

### Western blotting

Otic vesicles (HH18) were isolated and cultured, 30 otic vesicles from each condition were homogenized in ice cold Laemmli buffer with 50 mM dithiotreitol (DTT), Phosphatase Inhibitor Cocktail 2 and Protease Inhibitor Cocktail (both 1∶100, from Sigma-Aldrich). The homogenized samples were heated at 95°C for 5 min and frozen immediately. Gels were loaded with solutions containing equal amounts of proteins and the otic vesicle protein extracts were resolved by SDS-PAGE on 8% or 12% polyacrylamide gels. The proteins were transferred to nitrocellulose membranes and after incubation with blocking solution (5% non-fat dry milk in TRIS-buffered saline with 0.1% Tween-20: TBS-T), the membranes were probed overnight at 4°C with the appropriate specific primary antibodies to analyze the RAF kinases, pERK/ERK or pAkt/Akt [14] (See Supplementary material Table S1). All antibodies were diluted in blocking solution except anti-phospho-Akt antibody, which was diluted in TBS-T and 5% bovine serum albumin (BSA: Sigma-Aldrich, Saint Louis, MO). The membranes were subsequently washed and then incubated with the appropriate peroxidase-conjugated secondary antibody (1∶3000) for 1 h at RT. Antibody binding was visualized by chemiluminiscence (GE Healthcare, Buckinghamshire, UK) and exposed to X-ray film (Konica Minolta, Wayne, NJ). The films were scanned and the bands quantified by densitometry with Image J software (Wayne Rasband, National Institutes of Health, USA). At least three independent experiments were performed per condition and the statistical significance was estimated using the Student's t-test.

### Inmunohistochemistry

The sources, dilution, and cell specificities of the antibodies used for immunofluorescent staining are shown in Supplementary material Table 1. Samples were washed and permeabilized in 1% PBS/Triton-X-100 (PBS-T), and they were exposed to the primary antibodies overnight at 4°C. Non-specific binding sites were blocked for 1 h in PBS-T, 3% (wt/vol) BSA (Sigma-Aldrich) and 5% (vol/vol) normal goat serum.

For single immunostaining, sections were incubated for 2 h in a biotinylated anti-rabbit secondary antibody (1∶100, biotin-conjugated anti-rabbit, Chemicon), processed with ExtrAvidin-peroxidase conjugate solution (1∶200, Sigma). Finally, antibody binding was visualized using DAB as the chromogen and the sections mounted in Mowiol for observation under a Nikon 90i microscope.

For immunofluorescent staining of frozen sections, the primary antibodies were used as described above and the secondary antibodies were incubated for 2 h at room temperature. For dual-fluorescence immunolabeling, otic vesicles were incubated with Alexa Fluor 488 goat anti-mouse (1∶200), Alexa Fluor 647 goat anti-rabbit and/or Alexa Fluor 546 goat anti-rabbit secondary antibodies (1∶200; all from Molecular Probes, Eugene, OR). TxRed-conjugated phalloidin was used to identify the apical actin-containing structures of hair cells. Control experiments omitting the primary antibody were carried out to confirm that the staining patterns were specific for antigen recognition and additionally frozen sections from wild type or *B-Raf* ^−/−^ null embryos were included as negative controls (data not shown). The sections were mounted in Prolong Gold with DAPI (Invitrogen, Carlsbad, CA) and visualized by fluorescence (Nikon 90i, Tokyo, Japan) or confocal microscopy (Leica TCS SP2, Wetzlar, Germany). For whole-mount immunofluorescence, otic vesicles were incubated with the secondary antibodies for 3 h at room temperature, the otic vesicles were mounted in Vectashield with DAPI (Vector, Peterborough, UK) and the fluorescence was visualized by confocal microscopy (Leica TCS SP2, Wetzlar, Germany). At least five to six otic vesicles/frozen sections were analyzed for each condition from at least two independent experiments.

### BrdU incorporation and immunodetection

To study cell proliferation, otic vesicles were incubated with 5-Bromo-2′-deoxyuridine (Sigma-Aldrich, Saint Louis, MO), a thymidine analogue that is incorporated into DNA during the S phase of the cell cycle. BrdU (10 mg/ml) was added to the culture medium 1 h before the end of the incubation and its incorporation was detected with a specific antibody as above, but including a DNA denaturation step as recommended by the manufacturer (incubation in 50% (v/v) formamide-SSC, 40 minutes at 65°C and in HCl 2N, 30 minutes at 37°C, with a wash for 10 minutes in Tris 0.1M [pH 8]). At least five to six otic vesicles were assayed per condition in three independent experiments.

### *In situ* hybridization

*In situ* hybridization was performed on cryostat sections (20 µM) of specimens from HH24 embryos essentially as described previously with only minor modifications [13]. Digoxigenin-labeled sense and antisense RNA probes (1 mg/ml) were hybridized overnight at 72°C and their binding was detected by overnight incubation with an alkaline phosphatase-conjugated anti-digoxigenin antibody (1∶3500, Roche Applied Science), which was visualised with 5-Bromo-4-chloro-3-indolyl phosphate, nitro blue tetrazolium substrate (NTBT/BCIP, Roche Applied Science). The chicken C-*Raf* gene was amplified by PCR (*C-Raf* forward 5′-ACCTGCACGTTCAAGAGACC-3′; *C-Raf* reverse 5′-GCTACGAGCCTCTTCATTGC-3′) and subsequently ligated into a pGEM-T plasmid (Promega) to prepare the probe. Single-stranded sense (ApaI/T7) and antisense (PstI/Sp6) RNA probes were prepared by *in vitro* transcription and no specific signal was obtained when control sense probes were used (data not shown).

### Analysis of programmed cell death

The pattern of cell death in the otic vesicle was studied by Tdt-mediated dUTP nick-end labeling (TUNEL) of fragmented DNA using the kit *Dead-End™ Fluorometric TUNEL System* (Promega, Madison, WI) essentially as described by the manufacturer and adapted to whole organ labeling [14], [29]. The otic vesicles were mounted with Vectashield with DAPI (Vector) and visualized on a confocal microscope (Leica, TCS SP2). TUNEL-positive cells were counted using Image Analysis Software (Olympus, Tokyo, Japan) attributing a value of 1 to the control condition (no addition, 0S). At least five otic vesicles were assayed per condition in three independents experiments. The data are presented as the mean ± SEM and the statistical significance was estimated with the Student's t-test.

## Results

### RAF kinases are expressed during early inner ear development

**Figure 1:**
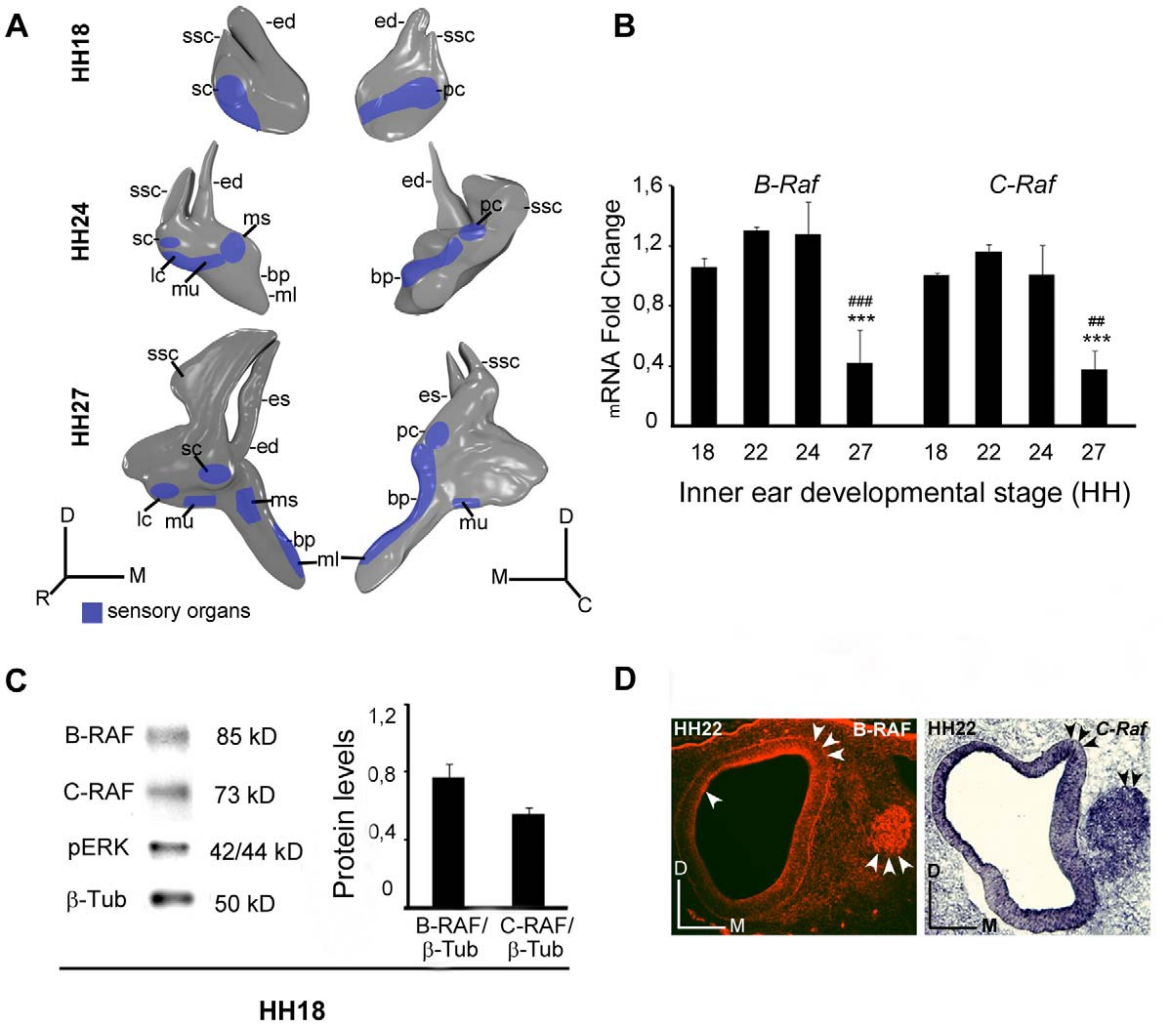
Expression of the B-RAF and C-RAF kinases during otic development. (**A**) Schematic drawings showing the development of the chicken inner ear at Hamburger and Hamilton stages HH18, HH24 and HH27. (**B**) Expression of inner ear *B-Raf* and *C-Raf* mRNA analyzed by qRT-PCR at different stages using Eukaryotic 18S rRNA as the endogenous housekeeping control gene. Gene expression was calculated as 2^−ΔΔCt^ and normalized to the levels at HH18. The results are expressed as the mean ± SEM of at least three independent experiments performed in triplicate. Statistical significance was estimated with the Student's t-test: ***P<0.005 versus HH18, ^##^P<0.01 versus HH24 and ^###^P<0.005 versus HH24. (**C**) HH18 otic vesicle lysates analyzed in western blots to determine the levels of B-RAF, C-RAF and phosphorylated ERK (pERK). ß-Tubulin (ß-Tub) was used as a loading control. A representative blot of three independent experiments is shown and the average densitometric measurements of the B-RAF and C-RAF bands are plotted as bars. The results are given as the mean ± SEM of three independent experiments. (**D**) Immunofluorescence of B-RAF and *in situ* hybridization of *C-Raf* at HH22 and HH24, respectively showing their location in the otic epithelium and acoustic-vestibular ganglion (arrowheads). Abbreviations: bp, basilar papilla; ed, endolymphatic duct; es, endolymphatic sac; lc, lateral crista; ml, macula lagena; ms, macula sacculi; mu, macula utriculi; pc, posterior crista; sc, superior crista; ssc, superior semicircular canal. Orientation: C, caudal; D, dorsal; M, medial; R, rostral.

The expression of chicken *B-Raf* and *C-Raf* at selected stages of otic development was studied by quantitative RT-PCR (Figure 1 A and B). The expression of transcripts encoding these RAF isoforms was comparable from HH18 to HH24, although the RNA transcripts for both these RAF kinases were strongly downregulated at stage HH27. Moreover, B-RAF and C-RAF proteins were both present in inner ear extracts from stage HH18 embryos, the stage at which otic vesicles can be explanted and cultured ex vivo (Figure 1 C). Indeed, there was a significant amount of phosphorylated ERK, a read out of RAF activity [30]. The presence of RAF kinases was further confirmed by immunohistochemistry for B-RAF and in situ hybridization for *C-Raf* (Figure 1 D) and the AVG was strongly stained for both RAF kinases. In addition, B-RAF expression was also evident in the otic epithelium (arrowheads in Figure 1 D).

### Spatiotemporal expression of B-RAF during inner ear development

**Figure 2:**
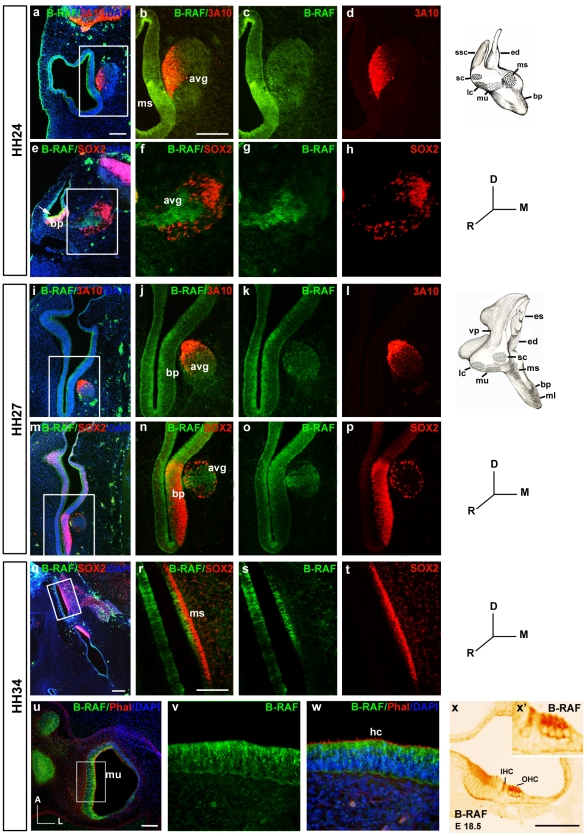
Spatiotemporal expression of B-RAF in the developing inner ear. **a**–**h**) At stage HH24, B-RAF (green throughout the figure) is abundantly expressed in the macula sacculi (ms) and in the acoustic-vestibular ganglion (avg), which is labelled red due to the expression of the axonal marker 3A10 (**a**–**d**). B-RAF is expressed strongly in the basilar papilla (bp, **e**, arrow). B-RAF and SOX2 (red), a transcription factor essential for the self-renewal of undifferentiated otic progenitors, are expressed in non-overlapping regions of the avg (**e**–**h**). **i**–**p**) At HH27, B-RAF is expressed strongly in the internal cell layers of the basilar papilla (bp, **i**–**l**), whereas SOX2 is present in more external cell layers (**m**–**p**). **q**–**w**) At HH34, SOX2 is expressed in supporting cells of the ms whereas B-RAF labels the hair cells (hc: **q**–**t**). TxRed-phalloidin staining (red) labels actin in the hc stereocilia of the macula utriculi (mu), and B-RAF is evident in the cytoplasm (**u**–**w**). B-RAF is also expressed in the outer (OHC) and inner hair cells (IHC) of E18.5 mouse embryos (**x**). **x′** shows a higher magnification of the sensory region in **x**. Schematic drawings of HH24 and HH27 inner ears are shown. The boxed areas show higher magnifications of the selected regions. Abbreviations: avg, acoustic-vestibular ganglion; bp, basilar papilla; ed, endolymphatic duct; es, endolymphatic sac; hc, hair cells; IHC, inner hair cells; OHC, outer hair cells; lc, lateral crista; ml, macula lagena; ms, macula sacculi; mu, macula utriculi; sc, superior crista; ssc, superior semicircular canal; vp, vertical canal pouch. Orientation: D, dorsal; M, medial; R, rostral. Scale bars: 100 µm.

In inner ear sections from HH24, HH27 and HH34 embryos, the distribution of B-RAF was determined by immunohistochemistry (Figure 2). The different cell types labeled were identified by staining for the neurofilament-related 3A10 protein [6] and SOX2, a transcription factor associated to immature pluripotent precursors. In mammals, SOX2 participates in the specification of the otic prosensory domain [31], [32] and the generation of cochlear neurons [33], while it is required for hair cell survival and regeneration in the inner ear of the zebrafish [34]. B-RAF was expressed homogenously in the otic epithelia and in the AVG at HH18 and HH22 (Figure 1 and data not shown), while its expression became more restricted as development proceeded. At HH24, B-RAF was abundantly expressed in the macula sacculi (Figure 2 a–d) and it was also expressed in the AVG, where a subset of neuroblasts was more strongly stained for B-RAF (Figure 2 e–h). B-RAF and SOX2 were expressed in adjacent regions and B-RAF expression did not appear to overlap with that of SOX2. A similar situation was also observed in the basilar papilla where even though both proteins appeared to overlap in some cells, the internal cells most strongly expressed B-RAF, whereas SOX2 expression was observed more laterally (Figure 2, arrow in e). At HH27, B-RAF expression was restricted to the internal layer of the otic epithelia and the cells of the AVG (Figure 2, i–p). At HH34, SOX2 was strongly expressed by supporting cells, whereas B-RAF was expressed strongly in the macula sacculi, limiting the region of SOX2 expression and suggesting that B-RAF is expressed by hair cells (Figure 2, q–t). To further explore the expression of B-RAF in hair cells, we used TxRed-phalloidin to label the actin in the stereocilia of hair cells (u, w). B-RAF expression was observed in the cytoplasm of both auditory and vestibular hair cells, and in AVG neurons (Figure 2, u–w, and data not shown). Specific hair cell expression of B-RAF was confirmed in E18.5 wild type mice, as was the specificity B-RAF by labeling null *B-Raf* mouse embryos (Figure 2, x–x′: Magariños, Rapp and Varela-Nieto, manuscript in preparation).

### The activity of RAF kinases is required for the proliferation and survival of otic neuroepithelial cells

**Figure 3:**
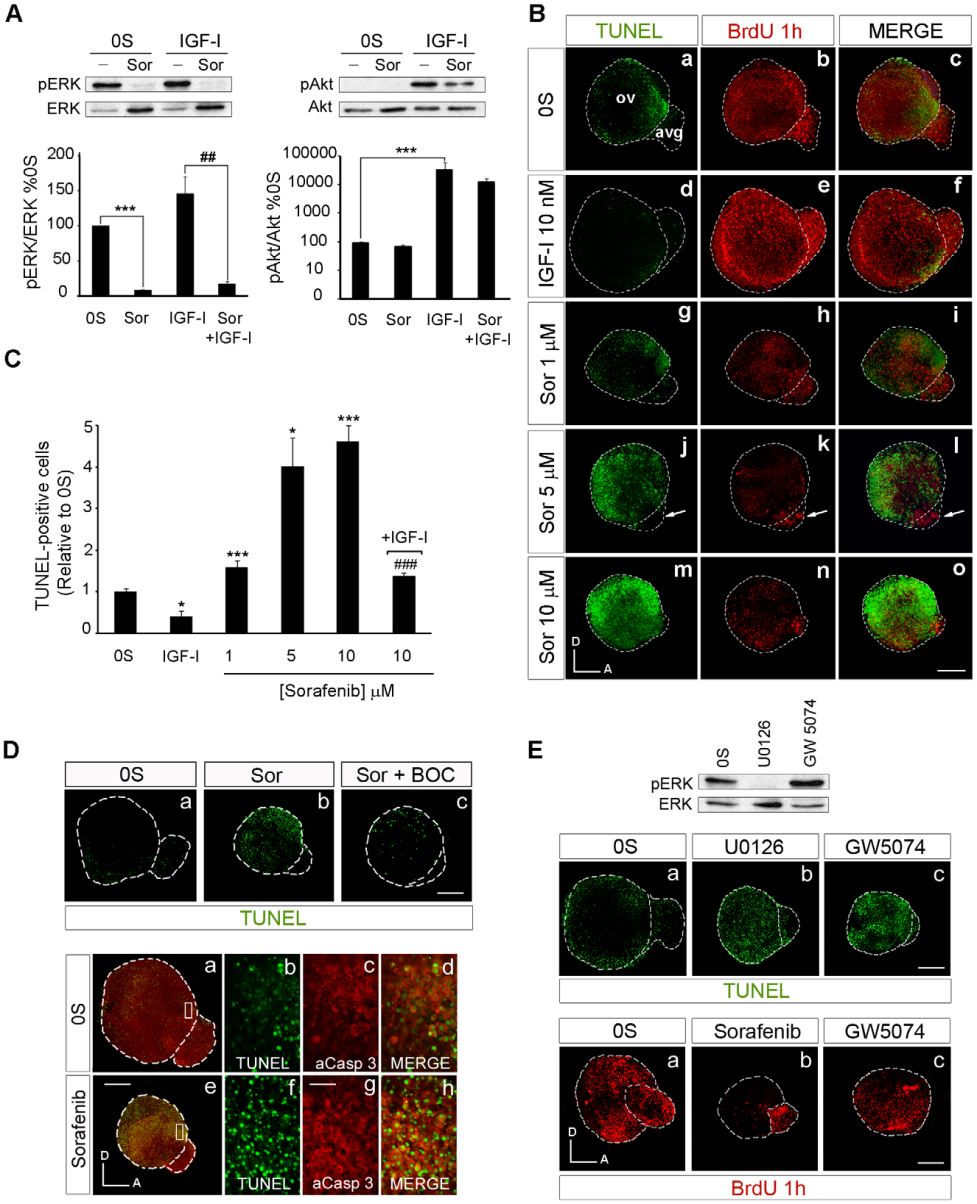
Selective inhibition of the RAF-MEK-ERK cascade blocks proliferation and promotes apoptosis. (**A**) **Sorafenib inhibits the RAF-MEK-ERK pathway.** Otic vesicles were explanted from stage HH18 chicken embryos and incubated for 24 h in serum-free medium (0S). The explants were then incubated for 1 h in serum-free medium without additives (0S), with IGF-I (10 nM), Sorafenib (Sor; 5 µM) or a combination of both IGF-I and Sorafenib. Otic vesicles were lysed and the levels of phosphorylated and unphosphorylated ERK and Akt kinases were quantified in Western blots by densitometry, as described in Materials and Methods. Representative blots are shown in the upper row. The results are expressed relative to the control value (0S), which was given an arbitrary value of 100, as the mean ± SEM of three independent experiments. Statistical significance was estimated with the Student's t-test: ***P<0.005 versus 0S and ^##^P<0.01 versus IGF-I. (**B**) **Apoptosis and proliferation in Sorafenib-treated cultures of otic vesicles.** Apoptotic cell death was visualized by TUNEL (green) in cultured otic vesicles. Proliferation was measured by the incorporation of BrdU (red) over 1 h. Otic vesicles were isolated from HH18 chicken embryos, made quiescent and cultured for 24 h in serum-free culture medium without additives (0S), with IGF-I (10 nM), Sorafenib (Sor; 1, 5 or 10 µM) or a combination of both IGF-I and Sorafenib. Scale bars, 150 µm. (**C**) **Cell death quantification of B.** The TUNEL positive nuclei were quantified relative to the 0S condition, which was given an arbitrary value of 1. The bars show the mean ± SEM of at least five otic vesicles from any of the conditions shown in B. Statistical significance was estimated with the Student's t-test: *P<0.05 versus control, ***P<0.005 versus control, ^###^P<0.005 versus Sorafenib 10 µM. (**D**) **Sorafenib increases cell death through a caspase-dependent mechanism.** Otic vesicles were isolated from HH18 chicken embryos and cultured for 24 h in serum-free culture medium without stimuli (0S; a upper panel), or cultured in the presence of Sorafenib 5 µM (Sor; b upper panel) or in combination with the pan-caspase inhibitor Boc-D-FMK 50 µM (Sor+BOC; c upper panel) and cell death was visualized using the TUNEL technique. Lower panel shows apoptotic cell death visualized by TUNEL staining (green) and immunostaining for activated-caspase-3 (red) of otic vesicles cultured in free serum (0S, a–d) or in the presence of Sorafenib 5 µM (e–h). Boxed areas in a and e are shown at a higher magnification to show the TUNEL-positive nuclei (b,f and merge) surrounded by activated caspase-3 (c,g and merge). Scale bar, 150 µm (a,e); 20 µm (b–d and f–h). (**E**) **Treatment of cultured otic vesicles with the MEK inhibitor U0126 and with the C-RAF inhibitor GW5074.** A representative blot of the effects of U0126 (50 µM) and GW5074 (1 µM) on ERK phosphorylation is shown. Apoptosis in cultured otic vesicles was visualized with TUNEL (upper panels, a–c). Proliferation was measured by the incorporation of BrdU (red) over 1 h in otocysts cultured with no additives (0S), with Sorafenib (5 µM) or with GW5074 (1 µM) (lower panels, a–c). Scale bar: 150 µm. Compiled projections of confocal microscopy images from otic vesicles are shown. A, anterior; D, dorsal. Abbreviations: AVG, acoustic-vestibular ganglion; OV, otic vesicle. The images shown are representative of at least three independent experiments, using five to six otic vesicles per condition.

The RAF-MEK-ERK phosphorylation cascade can be specifically inhibited by Sorafenib, an inhibitor of RAF kinase activity developed to treat B-RAF-associated cancer [16]. Otic vesicles were explanted and cultured ex vivo in the presence of Sorafenib to further understand the role of RAF activation in early inner ear development. This compound totally abolished ERK phosphorylation, both the basal phosphorylation and that induced by IGF-I (Figure 3 A, left panels). The specificity of Sorafenib to the RAF-MEK-ERK cascade was witnessed by its failure to effect Akt phosphorylation, both basal and IGF-I induced (right panels). Further insight into the actions of Sorafenib was obtained by studying its effects on cell proliferation and apoptosis in organotypic cultures of explanted HH18 otic vesicles. When cultured otic vesicles were exposed to Sorafenib, cell proliferation was reduced and apoptosis was induced in a dose-dependent manner (Figure 3 B). The number of apoptotic TUNEL positive cells found in the control otic vesicles (0S) was 2.5-fold higher than that found when IGF-I was added to the medium (Figure 3 C: [14]). However, the addition of increasing concentrations of Sorafenib (1, 5 and 10 µM) significantly increased the number of apoptotic cells by 1.8-, 4- and 4.6-fold, respectively (Figure 3 C and B, compare a with j and m). At the highest concentration of Sorafenib tested (10 µM), most cells in the otocyst were TUNEL positive (Figure 3 B, m). It is worth noting, that Sorafenib induced less programmed cell death in the AVG than in the otic vesicle epithelium, even though the size of the AVG decreased dramatically (Figure 3 B, arrow in j). The cell death was caspase-dependent as it was blocked by the pan-caspase inhibitor BOC (Figure 3 D, upper panels, a-c, and compare b with c). Apoptosis was further studied by combining TUNEL staining with the immunodetection of active caspase-3 (Figure 3 D, lower panels, a–h). The Sorafenib-treated otic vesicles showed areas of apoptotic cell death where TUNEL-labeled apoptotic nuclei were surrounded by cytoplasm containing active caspase-3 (Figure 3 D, lower panels, e–h).

As RAF activation leads to cell proliferation, we assessed bromodeoxyuridine (BrdU) uptake in cultured otic vesicles to measure the rate of proliferation. IGF-I promoted otic proliferation when compared with control cultures (Figure 3 B, compare b and e), while Sorafenib impaired BrdU incorporation in a dose dependent manner. As a consequence, the size of Sorafenib-treated otic vesicles was severely reduced (Figure 3 B, b, h, k, n). AVG size was also reduced, even though cell proliferation at the AVG was not strongly affected by Sorafenib (Figure 3 B, arrow in k), suggesting that neuronal cells that escaped from the RAF blockage are resistant to Sorafenib, possibly because RAF kinase activity is no longer required.

To confirm that the effects observed were a consequence of the inhibition of the RAF-MEK-ERK cascade a MEK inhibitor [35], U0126, was used (Figure 3 E). The effects of blocking RAF catalytic activity with Sorafenib were emulated by U0126, which abolished ERK phosphorylation (Figure 3 E), reduced cell proliferation (data not shown) and increased apoptosis (Figure 3 E, upper panels, compare a with b). In contrasts, treatment with the C-RAF highly specific inhibitor GW5074 [36] showed reduced AVG size, undifferentiated-rounded shape OV with reduced size and increased TUNEL positive cells (Figure 3 E, upper panels, compare c with a), but not evident changes in cell proliferation (Figure 3 E, lower panels, compare c with a). GW5074-treatment slightly reduced BrdU incorporation when compared to the 0S condition, whereas Sorafenib showed a more dramatic reduction on BrdU levels (lower panels in Figure 3 E, compare c with b). Furthermore, treatment with GW5074 increased ERK phosphorylation (Figure 3 E), suggesting that actions of C-RAF on apoptosis are, at least in part, independent of the activation of MEK and ERK.

These results show that inhibition of the RAF-MEK-ERK cascade caused a reduction in the rate of cell proliferation and an increase in the number of caspase-dependent apoptotic cells, without affecting Akt activity, thereby leading to a decrease in the number of neural progenitor cells.

### The activity of RAF kinases is required for otic neurogenesis

**Figure 4:**
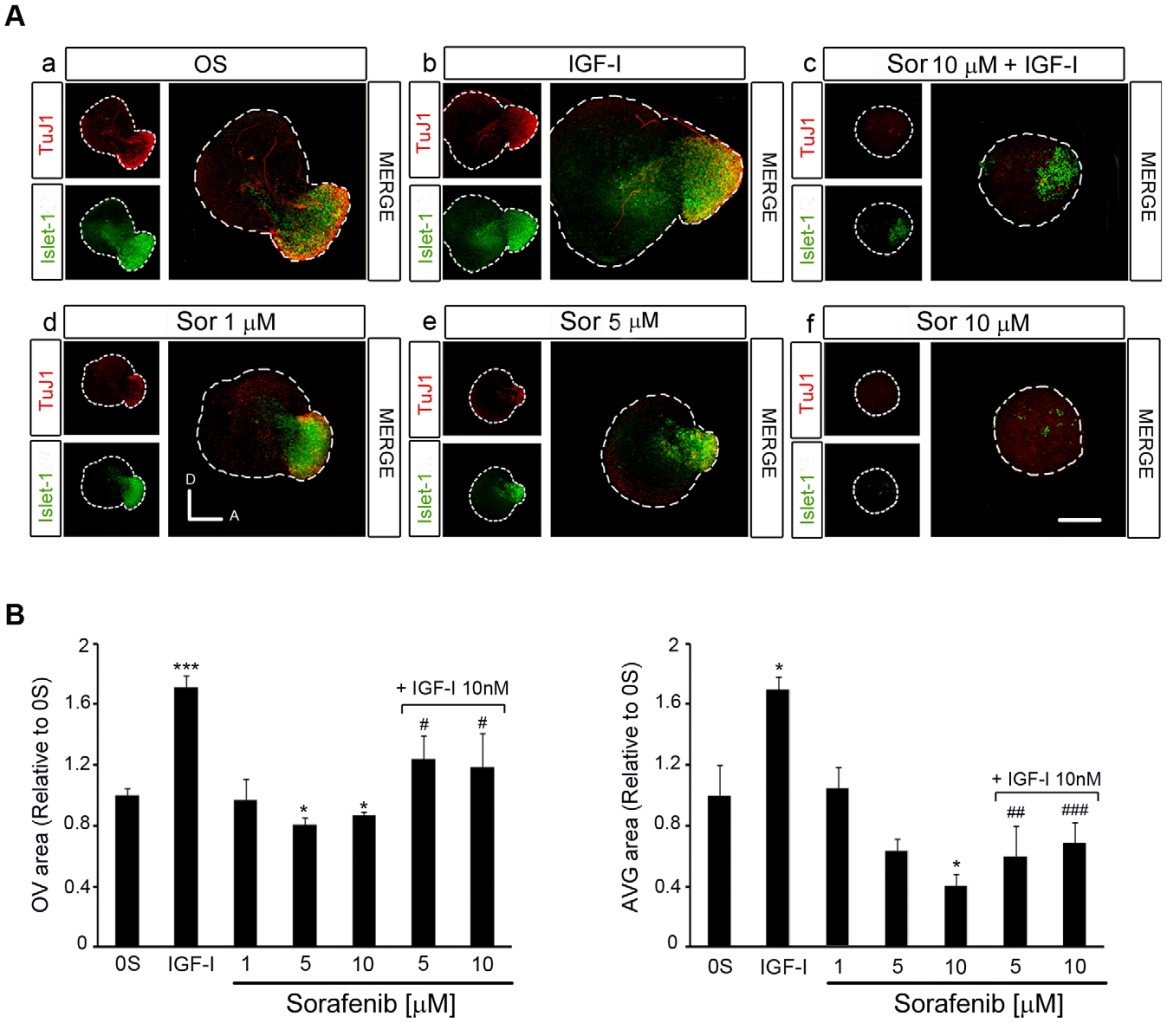
Inhibition of the RAF-MEK-ERK cascade impairs AVG formation. (**A**) Otic vesicles were isolated from HH18 chicken embryos and incubated for 24 h in serum-free culture medium without additives (0S), with IGF-I (10 nM), Sorafenib, (Sor;1, 5 or 10 µM) or a combination of Sor (10 µM) and IGF-I. Whole otic vesicles were then immunostained for the ganglion neuroblast nuclei marker Islet-1 (green) and for the marker of neural processes, TuJ1 (red). Fluorescence images were obtained from the compiled projections of confocal images of otic vesicles. Representative images of at least five to six otic vesicles per condition and from at least three independent experiments are shown. Orientation: A, anterior; D, dorsal. Scale bar: 150 µm. (**B**) The otic vesicles (OV) and the acoustic-vestibular ganglia (AVG) areas were measured with Image Analysis Software (Olympus, Tokyo, Japan). The data are expressed as the mean ± SEM relative to the control value (0S) and they were compiled from the analysis of at least five to six otic vesicles per condition. Statistical significance was estimated with the Student's t-test: *P<0.05, ***P<0.005 versus 0S; ^#^P<0.05, ^##^P<0.01 and ^###^P<0.005 versus IGF-I.

**Figure 5:**
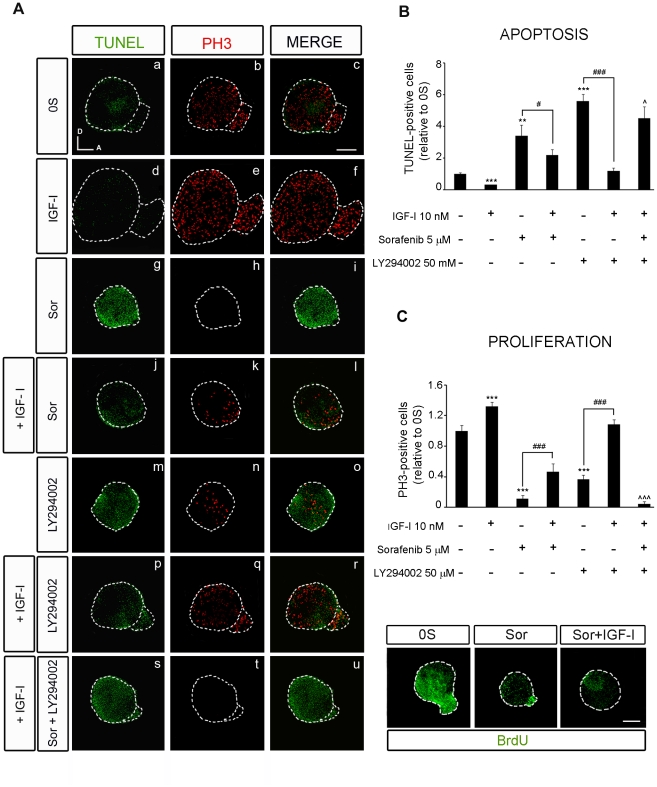
IGF-I partially rescues the effects of inhibiting RAF activity through the PI3K/Akt kinase pathway. (**A**) Apoptotic cell death was visualized by TUNEL (green) in cultured otic vesicles and proliferation was detected with the mitosis marker Phospho-Histone 3 (PH3, red). Otic vesicles were isolated from HH18 chicken embryos and cultured for 24 h in serum-free medium without additives (0S, a–c), with IGF-I (10 nM, d–f), Sorafenib, Sor, (5 µM, g–i), LY294002 (50 µM, m–o), a combination of IGF-I and Sor (j–l), IGF-I and LY294002 (p–r) or IGF-I, Sor and LY294002 (s–u). (**B**) TUNEL positive or (**C**) proliferative PH3-labeled cells were quantified as described in at least 5 otic vesicles per condition. The results are shown as the mean ± SEM relative to the 0S condition. Statistical significance was estimated with the Student's t-test: *P<0.05, ***P<0.005 versus 0S; ^#^P<0.05 and ^###^P<0.005 versus the indicated inhibitors; **∧**P<0.05, **∧∧∧**P<0.005 versus Sorafenib +IGF-I. Lower panels in **C** show BrdU (green) incorporation into cultured otic vesicles incubated for 24 h in the following conditions: 0S, with Sor (5 µM), or a combination of Sor and IGF-I (10 nM). Compiled projections of confocal images from otic vesicles are shown, and are representative of at least five to six otic vesicles per condition from three different experiments. Scale bar, 150 µm.

**Figure 6:**
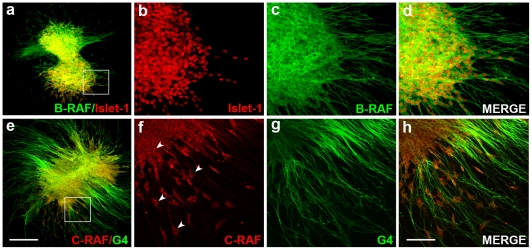
RAF proteins show different subcellular distribution in the acoustic- vestibular ganglion. AVG explants were obtained from stage HH19 chicken embryos and cultured in serum-free medium for 20 h with no additives (0S). (**a**–**d**) Whole AVG explants were immunostained for B-RAF (green) and Islet-1 (red) or (**e**–**h**) for C-RAF (red) and G4 (green), The cytoplasmatic distribution of C-RAF is shown (arrowheads). Fluorescence images were obtained from compiled projections of confocal images of AVG. Scale bar, 350 µm (a, e); 75 µm (b–d, f–h).

To further explore the regulation by IGF-I and the role of the RAF-MEK-ERK cascade in otic neurogenesis, the expression of the neuroblast markers Islet-1 and TuJ1 was analyzed in cultured otic vesicles treated with Sorafenib in the presence or absence of IGF-I [Figure 4, A; [6]]. The expression of Islet-1 and TuJ1 was reduced in a dose-dependent manner in the presence of Sorafenib, indicating that inactivation of the RAF kinase caused a loss of neuroblasts (Figure 4 A, compare a with d, e, f). Indeed, in otocysts exposed to Sorafenib, and hence with impaired RAF catalytic activity, there was a dose-dependent 20 and 40% reduction in the otic vesicle epithelia and AVG size, respectively (Figure 4 A) when compared to otocysts cultured under control conditions (Figure 4 B). The impact of RAF inactivation on the proliferation of otic precursors was further confirmed by studying the number of cells in mitosis and the incorporation of BrdU (Figure 5 A, C).

IGF-I promoted cell proliferation in the AVG neuroblast population, as witnessed by the increased number of Islet-1 positive cells and the 160% increase of the AVG area (Figure 4 A, compare a with b; Figure 4 B, bars on the right). TuJ1 labels neuroblasts at a more mature stage [4] and hence, TuJ1 positive cells are diminished or unaffected by the addition of IGF-I (Figure 4 A, compare a with b and c). IGF-I also promoted cell proliferation in the otic epithelium (Figure 5 A, compare b with e). In the presence of IGF-I, the number of Islet-1 positive cells and mitosis increased in Sorafenib-treated otic vesicles (Figure 4 A, compare c with f), while apoptosis was reduced (Figure 5 A–C), leading to a small but significant recovery of the OV and AVG (Figure 4 B). Because Sorafenib completely blocks IGF-I activation of the RAF-MEK-ERK pathway (Figure 3), these results suggest that the rescue of the neuroblast population by IGF-I is mediated by an alternative pathway.

### IGF-I-induction of the PI3K/Akt kinase pathway rescues otic progenitors from apoptosis

IGF-I activates the Akt pathway even in the presence of Sorafenib and the total inactivation of the RAF-MEK-ERK cascade (Figure 3). To further define the roles of the RAF-MEK-ERK and PI3K/Akt kinase pathways in otic neurogenesis, we studied the proliferation and apoptosis of explanted otic vesicles treated with combinations of RAF and Akt inhibitors in culture. Cell death was studied by detecting the TUNEL positive cells (TUNEL) and cell proliferation was detected by following the nuclei labeled with phospho-histone-3 (PH3), which identifies cells in the M phase of the cell cycle (Figure 5 A), or by BrdU incorporation (Figure 5 C).

LY294002 is a well-characterized inhibitor of the PI3K/Akt kinase pathway that specifically impairs Akt phosphorylation [37]. Inhibition of the PI3K/Akt kinase pathway increased the number of apoptotic cells (5.5-fold) and reduced the amount of cells expressing the mitotic marker PH3 (0.7-fold: Figure 5 A, m–o, and bars in B and C). The presence of IGF-I partially impaired the effects of LY294002 as the number of proliferating cells in treated otic vesicles was similar to that of the controls (Figure 5 A, p–r, and bars in B and C). In combination, Sorafenib and LY294002 completely abolished the effects of IGF-I on proliferation and apoptosis (Figure 5 A, s–u). TUNEL staining was stronger in vesicles exposed to Sorafenib and LY294002 than in those treated with Sorafenib alone (1.3-fold) or those exposed to Sorafenib and IGF-I (2.0-fold: Figure 5 B). The rate of proliferation was also reduced 3-fold in the presence of Sorafenib plus LY294002 when compared to Sorafenib alone, and 12-fold when compared to Sorafenib plus IGF-I (Figure 5 C). These results indicate that the protective effects of IGF-I on survival when the RAF-MEK-ERK cascade is inhibited are mediated by the induction of the PI3K/Akt kinase pathway. In the presence of IGF-I, Sorafenib did not completely abolish mitosis (Figure 5 C, quantification of PH3 positive cells) but it did abolish the incorporation of BrdU (Figure 5 C, lower panels), suggesting that IGF-I can partially overcome the effects of RAF inhibition. These data possibly reflect the capacity of IGF-I to sustain progenitors that are already in the M-phase of the cell cycle but not to promote cell cycle entry.

### RAF activity is required for neuronal progenitor cell differentiation and the outgrowth of processes from sensory otic neurons

**Figure 7:**
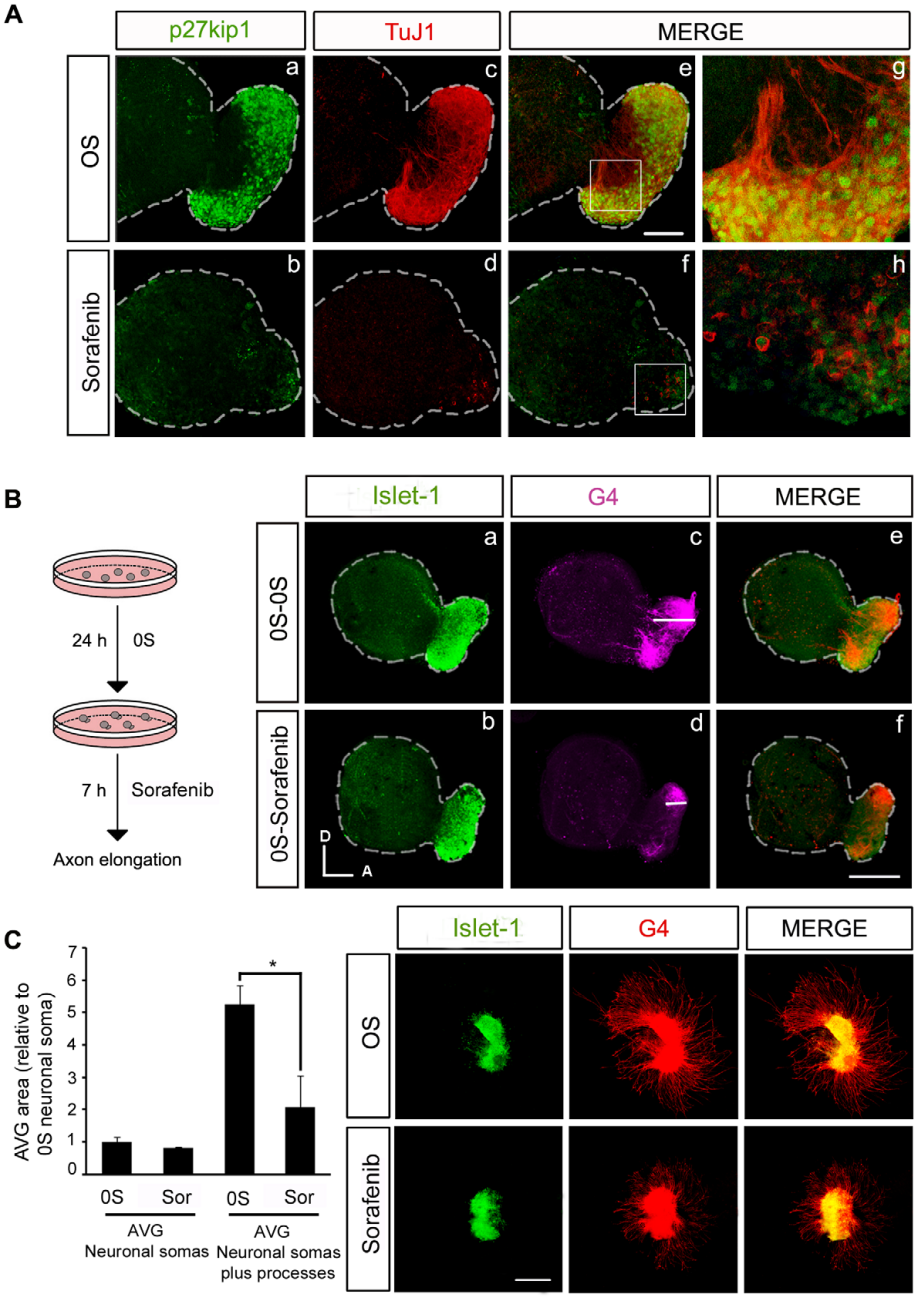
RAF kinase activity is required for the correct outgrowth of sensory otic neuron processes. (**A**) Otic vesicles were isolated from HH18 chicken embryos and incubated for 24 h either in serum-free medium without additives (0S, a,c,e,g) or in the presence of Sorafenib (2.5 µM) (b,d,f,h). Immunohistochemistry of whole otic vesicles was carried out by double-staining for the nuclear cyclin-dependent kinase inhibitor p27^kip1^ (green) and for the marker of neural processes, TuJ1 (red). The boxed areas in panels e and f, correspond to the enlarged images in panels g and h respectively. Scale bar: 75 µm. (**B**) Otic vesicles were isolated from HH18 chicken embryos and incubated for 24 h in serum-free medium as in the 0S condition in A, and they were then incubated for a further 7 h without additives (0S-0S, a,c,e) or with Sorafenib (2.5 µM: 0S-Sorafenib, b,d,f). Whole otic vesicles were immunostained for the ganglion neuroblast nuclei marker, Islet-1 (green), and for the G4-glycoprotein marker of neuronal processes (G4, magenta). Note the differences in the magnitude of the white bars in the region in the acoustic-vestibular ganglia corresponding to the staining of neural processes in panels c and d. Scale bar: 150 µm. (**C**) Acoustic-vestibular ganglia (AVG) explants were obtained from stage HH19 chicken embryos and cultured in serum-free medium for 20 h with no additives (0S) or with Sorafenib (2.5 µM). Whole AVG explants were immunostained for G4 (red) and Islet-1 (green). Sorafenib-treated AVG have shorter processes. Scale bar: 300 µm. Fluorescence images were obtained from compiled projections of confocal images of otic vesicles and AVG. Bar graph on the left shows the quantification of the neuronal soma area in the AVG, which does not vary following Sorafenib treatment. In contrast, there is a statistically significant difference in the area of the AVG covered by processes (*P<0.05, Sorafenib versus 0S). Representative images of three independent experiments using five to six otic vesicles or AVG per condition are shown. Orientation: A, anterior; D, dorsal.

B-RAF has previously been reported to play a role in sensory axon and dendrite growth. Figure 6 shows B-RAF and C-RAF expression in explanted AVG cultured in serum-free medium. B-RAF was highly expressed in the cytoplasm and in the neural processes (Figure 6, a–d), whereas C-RAF showed a more restricted cytoplasmic expression (Figure 6, e–h, arrowheads). To study the role of RAF kinases in neural process outgrowth we examined the differentiation state of AVG cultured explants. Postmitotic otic neurons were identified by labeling with the nuclear cyclin-dependent kinase inhibitor p27^kip1^ [13] and with TuJ1, a neural tubulin that is found in processes (Figure 7 A). AVG neurons that have exited the cell cycle and that are located more distally with respect to the neurogenic zone of the otic vesicle epithelium expressed p27^kip1^ (Figure 7 A, a), and their TuJ1 staining indicated that they had begun to extend axons towards the otic vesicle (Figure 7 A, c, e, g). Sorafenib treatment of cultured otic vesicles reduced the number of neuroblasts and mature neurons (Figure 7 A, compare a and b) and more interestingly, these mature neurons did not develop axons since their TuJ1 expression remained surrounding the cytoplasm (Figure 7 A, d, f, h). These results suggested that RAF-MEK-ERK signaling is necessary to initiate axonal growth.

To determine whether RAF activity is required for axonal growth once differentiation has been initiated, axonal growth was followed with the immature neuroblast marker Islet-1 and the axonal glycoprotein G4 [6]. Otic vesicles were cultured for 24 h to allow AVG formation, and the elongation of the processes was allowed to continue for 7 h in the presence (0S- Sorafenib) or absence of Sorafenib (0S-0S: Figure 7, B). After 24 h, mature otic neurons have already started to extend axons to innervate the dorsal (vestibular) and ventral (auditory) sensory epithelia (Figure 7 B, c and e). However, subsequent exposure to Sorafenib impaired this process and the axons remained shorter than those of controls (Figure 7 B, compare white bar in c with that in d), both in the otic vesicle and AVG areas (Figure 7 B, compare a and e with b and f). Similar experiments were then performed on isolated explanted AVG to further study the role of RAF-MEK-ERK signaling in otic neuronal differentiation. Again, exposure to Sorafenib (2.5 µM) caused a dramatic reduction in the number and length of processes (Figure 7 C), despite affecting the size of the AVG. When the areas of the AVG with neuronal soma alone or plus processes were quantified it was confirmed that RAF activity is required for otic neuron maturation and the outgrowth of processes.

## Discussion

Inner ear organogenesis requires strict spatial and temporal regulation of cellular proliferation, death and differentiation to generate the appropriate number of different cell types and their interconnections [2]. IGF-I drives cell proliferation and the survival of otic progenitors, and it is essential for neuronal differentiation in the time window between neuronal cell fate specification and neurotrophin dependence [4]. RAF proteins are serine/threonine kinases that regulate the RAF-MEK-ERK signaling pathway involved in the transduction of extracellular stimuli into cellular responses [16]. Three RAF kinase isoforms exist in mammals, A-, B- and C-RAF, whose activity is exquisitely regulated at the post-transcriptional level by a number of different mechanisms [38]. C-RAF activation is regulated by IGF-I and it is essential for cell proliferation in the otic vesicle [15]. However, no studies have been conducted on B-RAF even though this isoform is more active as a protein kinase and more abundantly expressed in the nervous system, where it is fundamental for axonal and dendrite growth [25], [27], [39], [40].

Here we show that B-RAF and C-RAF transcripts are expressed during inner ear development in a specific spatiotemporal pattern and that B-RAF is expressed specifically in neurosensorial components of the inner ear at later stages of development. RAF transcripts are translated into proteins that phosphorylate ERK, and RAF activity is regulated in the inner ear by IGF-I. Such RAF-MEK-ERK signaling is required for neuroepithelial cell proliferation and survival in this structure, although IGF-I can restore cell survival by activating the PI3K/Akt kinase pathway when RAF-MEK-ERK is inhibited by Sorafenib. However, IGF-I it is not able to restore cell proliferation in these conditions. Finally, we demonstrate that RAF kinase activity is required for neuronal progenitor cell differentiation and for the outgrowth of sensory otic neuron processes.

B-RAF and C-RAF are expressed in a similar temporal pattern from HH18 to HH27, when the striking reduction in both transcripts suggests that the transcription of both RAF isoforms is developmentally regulated. Up to stage HH24 these kinases are expressed homogeneously in the otic epithelia and AVG, and during these stages RAF kinases support the basic cellular programmes of otic progenitors. As development proceeds, the activity of RAF kinases may be more directly related to differentiation and post-differentiation events, and accordingly they become more spatially restricted. At HH27 when auditory and vestibular hair cells have just differentiated, both RAF kinases are down-regulated and the strongest B-RAF expression is associated to sensory hair and neuronal cells. Indeed, RAF kinases participate in late differentiation processes of other cell types such as T-cells [41] and sensory cells [42], as well as in post-differentiation events, such as cortical neuron migration [20] or the modulation of synaptic plasticity [43].

Due to the established role of RAF kinases in cancer, the search for inhibitors of RAF-MEK-ERK signaling has been intense [16]. Sorafenib 43-9006 is a potent small-molecule that inhibits RAF kinases, and its use has been approved for renal carcinoma therapy, as well as in clinical trials for melanoma and thyroid cancer [44]. Sorafenib was primarily identified as a C-RAF inhibitor but upon further characterization, it was shown to inhibit B-RAF and other kinases involved in angiogenesis [24]. We have used Sorafenib to study the influence of RAF kinases on otic progenitors and their regulation by IGF-I. Our results show that Sorafenib effectively inhibits basal and IGF-I induced ERK phosphorylation, without affecting Akt phosphorylation. Inhibition of RAF catalytic activity by Sorafenib also caused an increase in caspase-dependent apoptosis. Interestingly, in some melanoma cell lines this is not the case and Sorafenib-dependent apoptosis is caspase independent [45]. RAF activation of mitochondrial targets such as BAD [21], [46], ASK-1 [47], and MST-2 [48] has an anti-apoptotic effect and therefore, RAF inactivation can provoke cell death. RAF inactivation also annuls the activity of the MEK-ERK module, which along with the lack of activation of cell cycle proteins and transcription factors, such as retinoblastoma [49], Cdc25 [50] or AP1 [51], [52], may also cause apoptosis of proliferating cells. MEK inhibition by U0126 [35] and C-RAF inhibition by GW5074 [36] also caused apoptosis but presented different traits. The MEK inhibitor completely abolished ERK phosporylation and, in contrast, GW5074 induced it. Paradoxical actions of C-RAF inhibitors have been reported in other neuronal contexts [53], [54] and elimination of C-RAF activity by either knocking it out or siRNA targeting did not altered ERK phosphorylation [55], [56]. Therefore, RAF quinases inactivation could promote apoptosis of otic progenitor cells by both ERK dependent and independent mechanisms, as reported in other cell types [57].

The inactivation of the RAF pathway with Sorafenib also produced a dramatic decrease in cell proliferation since the RAF-MEK-ERK cascade plays a fundamental role in the G1/S transition, where its signaling induces cyclin D1 and down-regulates many other antiproliferative genes [52]. Accordingly, exposing cultured otic vesicles to Sorafenib caused a dose-dependent decrease of BrdU incorporation at S phase. U0126 treatment also reduced proliferation in the otic vesicle; however, the specific inhibition of C-RAF with GW5074 did not show the striking reduction on proliferation observed with the other inhibitors. In mice, of the three RAF isoforms, C-RAF appears to be preferentially involved in promoting survival, rather than controlling proliferation [55], [58]. In contrast, B-RAF is essential for ERK activation [25] that in turn triggers cell proliferation [59]. These data suggest that RAF kinases also have distinct roles during chicken inner ear development. C-RAF would preferentially promote anti-apoptotic signaling whilst B-RAF, through the MEK-ERK module, would modulate proliferation of neuroepithelial progenitors.

Experiments with IGF-I showed that RAF activity is essential for the progression of cell proliferation but not for cell survival. Indeed, IGF-I was even able to rescue otic progenitors by activating the PI3K/Akt pathway in the presence of Sorafenib. Blockage of the PI3K/Akt kinase pathway with LY294002 indicated that IGF-I is dependent on Akt activation for cell survival. Therefore, IGF-I orchestrates cell proliferation and survival in the otic vesicle through distinct pathways, although cross-talk between signaling pathways also occurs, as reported in other cell contexts [60].

The AVG is generated from a pool of neuroepithelial progenitors that when specified in the otic vesicle epithelia, migrate from the neurogenic zone and form the ganglia [4]. C-RAF and B-RAF are expressed in otic neurons but they exhibit distinct subcellular distribution, as reported in the rat brain [43]. B-RAF is abundantly expressed in cell bodies and neuronal processes, while C-RAF expression is more restricted to the cytoplasmic compartment. Exposure to Sorafenib caused a dramatic decrease in the area of the AVG, although inhibiting RAF kinase activity did not appear to affect the population of neuronal cells in the AVG, which continued to proliferate and exhibited little apoptosis. These data suggest that mature neurons do not require RAF kinases for survival but that RAF activity is essential for early neurogenesis. Accordingly, Sorafenib caused a clear reduction of mature neurons as indicated by the reduced levels of axonal outgrowth markers. This observation suggested that the RAF-MEK-ERK cascade may be involved in differentiation of neural cells in the AVG, as seen in the differentiation of cortical and dorsal neurons [42], [40]. Otic neuronal identity and axonal growth are determined by various factors once the cell has exited the cell cycle [4]. Axonal growth in post-mitotic p27^kip1^ positive AVG neurons was almost completely inhibited in the presence of Sorafenib, indicating that RAF kinase activation plays a fundamental role in the late differentiation of otic neurons.

In summary, we show here that B-RAF and C-RAF are expressed during chicken inner ear development in specific spatiotemporal patterns, and that RAF-MEK-ERK signaling is required for neuroepithelial cell proliferation and otic neuronal differentiation.

## Supporting Information

Table S1 Primary antibodies. 1: Antibody type: RbP, rabbit polyclonal; MouM mouse monoclonal; GtP goat polyclonal. 2: Technique: IHF, Immunohistofluorescence. WB, Western Blotting 3: Monoclonal antibody developed by Thomas Jessell and Jane Dodd were obtained from the Developmental Studies Hybridoma Bank developed under the auspices of the NICHD and maintained by the University of Iowa, Department of Biological Sciences, Iowa City, IA 52242.
(0.04 MB DOC)
