# Supplementary material for: RAF Kinase Activity Regulates Neuroepithelial Cell Proliferation and Neuronal Progenitor Cell Differentiation during Early Inner Ear Development
Source: PLoS One. 2010 Dec 28;5(12):e14435. doi: 10.1371/journal.pone.0014435 (PMC3010996; doi:10.1371/journal.pone.0014435)
Supplement: Table S1 — Primary antibodies. 1: Antibody type: RbP, rabbit polyclonal; MouM mouse monoclonal; GtP goat polyclonal. 2: Technique: IHF, Immunohistofluorescence. WB, Western Blotting 3: Monoclonal antibody developed by Thomas Jessell and Jane Dodd were obtained from the Developmental Studies Hybridoma Bank developed under the auspices of the NICHD and maintained by the University of Iowa, Department of Biological Sciences, Iowa City, IA 52242. (0.04 MB DOC) [file pone.0014435.s001.doc]

**Table S1**

| **ANTIGEN** | **TYPE** ^1^ | **SOURCE/Cat. no** | **CONC.** | **TECHNIQUE^2^** |
| --- | --- | --- | --- | --- |
|  |  |  |  |  |
| Islet-1 ^3^ | MouM | DSHB/ 39.4D5 (*supernatant*) | 1:3 | IHF |
| Tuj-1 (ß-III Tubulin) | RbP | Covance (Berkeley, CA)/ PRB-435P | 1:1000 | IHF |
| C-RAF  B-RAF  B-RAF  SOX2 | MouM    RbP  RbP  GtP | BD Transduction Laboratories/ 610151  Santa Cruz/ SC-9002  Santa Cruz/ SC-166  Santa Cruz/ SC-17320 | 1:500  1:10  1:1000  1:100  1:50 | WB  IHF  WB  IHF  IHF |
| 3A10 ^2^ | MouM | DSHB/ 3A10 (*supernatant*) | 1:10 | IHF |
| BrdU | MouM | Sigma-Aldrich/ B8434 | 1:150 | IHF |
| p27^kip1^  ß-Tubulin | MouM  MouM | BD Transduction Laboratories/ 610241  Sigma-Aldrich/ T4026 | 1:50  1:1000 | IHF  WB |
| ERK | RbP | Cell Signalling/ 9102 | 1:1000 | WB |
| pERK | RbP | Cell Signalling/ 9101 | 1:1000 | WB |
| Akt1/2 | GtP | Santa Cruz/ SC-1619 | 1:1000 | WB |
| phospho-Akt^Ser473^ | RbP | Cell Signalling/ 9271 | 1:1000 | WB |
| Phospho-Histone 3 | RbP | Upstate/ 06-570 | 1:100 | IHF |
| G4 glycoprotein  Active Caspase-3 (aCasp 3) | RbP  RbP | [51-52]  Promega/G7481 | 1: 500  1:30 | IHF  IHF |
|  |  |  |  |  |

**Table 1. Primary antibodies**

1: Antibody type: RbP, rabbit polyclonal; MouM mouse monoclonal; GtP goat polyclonal.

2: Technique: IHF, Immunohistofluorescence. WB, Western Blotting

3: Monoclonal antibody developed by Thomas Jessell and Jane Dodd were obtained from the Developmental Studies Hybridoma Bank developed under the auspices of the NICHD and maintained by the University of Iowa, Department of Biological Sciences, Iowa City, IA 52242.
